# Hypothalamic BOLD response to glucose intake and hypothalamic volume are similar in anorexia nervosa and healthy control subjects

**DOI:** 10.3389/fnins.2015.00159

**Published:** 2015-05-05

**Authors:** Anna M. van Opstal, Anna M. Westerink, Wouter M. Teeuwisse, Mirjam A. M. van der Geest, Eric F. van Furth, Jeroen van der Grond

**Affiliations:** ^1^Department of Radiology, Leiden University Medical CenterLeiden, Netherlands; ^2^Center for Eating Disorders Ursula, RivierduinenLeiden, Netherlands; ^3^Department of Psychiatry, Leiden University Medical CenterLeiden, Netherlands

**Keywords:** anorexia nervosa, case control study, glucose sensing, functional MRI, hypothalamus, voxel based morphomerty

## Abstract

**Background:** Inconsistent findings about the neurobiology of Anorexia Nervosa (AN) hinder the development of effective treatments for this severe mental disorder. Therefore, the need arises for elucidation of neurobiological factors involved in the pathophysiology of AN. The hypothalamus plays a key role in the neurobiological processes that govern food intake and energy homeostasis, processes that are disturbed in anorexia nervosa (AN). The present study will assess the hypothalamic response to energy intake and the hypothalamic structure in patients with AN and healthy controls.

**Methods:** Ten women aged 18–30 years diagnosed with AN and 11 healthy, lean (BMI < 23 kg/m^2^) women in the same age range were recruited. We used functional magnetic resonance imaging (MRI) to determine function of the hypothalamus in response to glucose. Structural MRI was used to determine differences in hypothalamic volume and local gray matter volume using manual segmentation and voxel-based morphometry.

**Results:** No differences were found in hypothalamic volume and neuronal activity in response to a glucose load between the patients and controls. Whole brain structural analysis showed a significant decrease in gray matter volume in the cingulate cortex in the AN patients, bilaterally.

**Conclusions:** We argue that in spite of various known changes in the hypothalamus the direct hypothalamic response to glucose intake is similar in AN patients and healthy controls.

## Introduction

Anorexia nervosa (AN) is a severe mental disorder mostly seen in teenage girls and young women. Individuals with AN are preoccupied with food and eating rituals to the point of obsession, engage in relentless restrictive eating and compulsively over-exercising, often become severely emaciated and have a distorted body image (American Psychiatric Association, 2000). The disorder is characterized by high rates of chronicity, morbidity and mortality (Papadopoulos et al., 2009). Although it was long thought that AN was primarily caused by psychosocial factors, recently many different neurobiological factors have been shown to be involved in the pathology of AN. Inconsistent findings hinder our understanding of this complex disorder and thereby the development of effective treatments (Kaye et al., 2013). Therefore, it is important to gain a better understanding of the neurobiological factors underlying AN.

A brain region that seems to be an important point of focus for neurobiological AN research is the hypothalamus, as it plays a key role in the intricate and complex neuroendocrine interactions that govern food intake and energy homeostasis. Hypothalamic functioning is known to be affected in AN; the hypothalamic-pituitary-adrenal (HPA) axis is hyperactive (Lawson et al., 2013) and there is a dysregulation of neuropeptides involved in the regulation of food intake and satiety (Bailer and Kaye, 2003; Smitka et al., 2013). The function of the HPA axis in AN has been extensively studied. However, to the best of our knowledge, the hypothalamic response to energy intake has not been investigated in AN. Using functional magnetic resonance imaging (fMRI) it is possible to investigate the hypothalamic response to energy intake by measuring the direct activity of the hypothalamus during nutrient ingestion. Using this technique in healthy humans, an oral glucose load shows a decrease in hypothalamic neuronal activity, (Vidarsdottir et al., 2007; Teeuwisse et al., 2012) whereas no such response was observed in patients with type 2 diabetes (Vidarsdottir et al., 2007). Furthermore, a delayed and attenuated response is found in obese participants (Matsuda et al., 1999). This indicates that in these patients, the hypothalamus inappropriately perceives and/or processes signals in response to a nutrient load, possibly reflecting an abnormal perception of the current metabolic status. Due to the known changes in hypothalamic functioning, patients with AN might also show different hypothalamic neuronal activity in response to an oral glucose load compared to healthy participants. Earlier studies assessed hypothalamic activation using fMRI before and after a meal in AN patients, but not directly during a nutrient load (Holsen et al., 2012). As the hypothalamic response to nutrients and subsequent feeding behavior is quite fast, it will be even more informative to see the direct response to a nutrient load. In addition to changes in the function of the hypothalamus several studies on psychiatric disorders also show abnormalities in hypothalamic structure (Goldstein et al., 2007). A decreased volume of the hypothalamus is also found in other disorders with a similar dysfunction of the HPA axis as in AN (Terlevic et al., 2013).

The aim of the present paper is to further elucidate hypothalamic functioning and structure in AN by fMRI and volume calculations of the hypothalamus together with a voxel-wise analysis to detect differences in local gray matter volume. We hypothesize that the hypothalamic neuronal activity after a glucose load might be different in female AN patients compared to healthy female control participants. Subsequently, we hypothesize that AN patients might have a smaller hypothalamic volume compared to healthy controls.

## Methods

### Participants

We recruited ten Caucasian women aged 18–30 years from Center for Eating Disorders Ursula, diagnosed with AN according to the Diagnostic and Statistical Manual of Mental Disorders Fourth edition criteria, (American Psychiatric Association, 2000) with a body mass index (BMI) < 17.5 kg/m^2^. 11 Healthy and lean (BMI < 23 kg/m^2^) Caucasian women in the same age range were recruited as control participants via advertisements at the University of Leiden. A standard MRI screening form and a health and lifestyle questionnaire were used to assess eligibility. Exclusion criteria for both patients and controls were: diabetes mellitus (DM) or a history of DM in first grade relatives, chronic diseases, genetic, or somatic disease affecting the brain, substance abuse, or addiction according to DSM-IV criteria (except smoking) and MRI contraindications. In addition, control participants who were on a weight reducing diet, lost, or gained more than 3 kg in weight over the last 2 months and/or who did not use oral contraceptives were excluded from participation. The latter criterion was set to minimize influence of menstrual cycle and to ensure stable levels of female hormones. The study protocol was approved by the local institutional review board, and written informed consent was obtained from all participants. To compare demographic data between the AN patients and controls independent means *t*-tests were used.

### Data acquisition

Participants were asked to fast (no food or beverage except water) from 10 p.m., the night before the MRI scanning. The MRI was performed between 8.30 and 10.45 a.m., To overcome the feeling of anxiety and to increase willingness to participate in our study, AN patients were allowed to skip breakfast and their morning snack on the study day in exchange for drinking the glucose solution. All participants received a glucose solution during the scanning session consisting of 50 g of glucose dissolved in 200 ml water. This was administered during the hypothalamus fMRI scan via a tube attached to the head coil of the scanner. After the scanning session participants' height and weight were measured.

MRI was performed at our institution using a 3.0 Tesla Achieva clinical scanner (Philips Healthcare, Best, The Netherlands). The protocol comprised of a whole brain 3DT1 (repetition time 9.8 ms, echo time 4.6 ms, field of view 224 × 177 × 168 mm, voxel size 1.17 × 1.17 × 1.20 mm, scan time 4.55 min) for planning and two single-slice, mid-sagittal scans: a T1-weighted Turbo Spin Echo sequence for imaging mid-sagittal anatomical structures (repetition time 550 ms, echo time 10 ms, field of view 208 × 208 mm, voxel size 0.52 × 0.52 × 14 mm, scan time 1.15 min) and a T2^*^-weighted BOLD-fMRI echo-planar imaging sequence (repetition time 120 ms, echo time 30 ms, flip angle 30°, field of view 208 × 208 mm, voxel size 0.81 × 0.81 × 14 mm, scan time 30 min, 707 time points). A slice thickness of 14 mm was chosen to incorporate the hypothalamus in the left to right direction and to ensure an adequate signal-to-noise ratio and modulus and phase images were acquired to enable complex averaging in post-processing. 8 min after the start of the fMRI scan, participants were signaled to drink the glucose solution.

### MRI data analysis

Both the fMRI and anatomical scans were processed and analyzed using different tools of FMRIB Software Library (FSL). (Smith et al., 2004; Woolrich et al., 2009; Jenkinson et al., 2012) Statistical analyses were performed using SPSS version 20 (SPSS Inc., Chicago IL, USA). All 707 functional images of each time series were motion corrected by registration to the image that was taken in the middle of the post-drink period (± image 450) with the Multimodality Image Registration using Information Theory software by maximization of mutual information (Maes et al., 1997). The resulting registration matrix was applied to the real and imaginary images that were calculated from the original phase and modulus images. Complex data were averaged for each set of four subsequent volumes to reduce phase artifacts caused by head motion or swallowing. Modulus images were then recalculated rendering 177 movement and phase-error corrected images.

**Figure 1:**
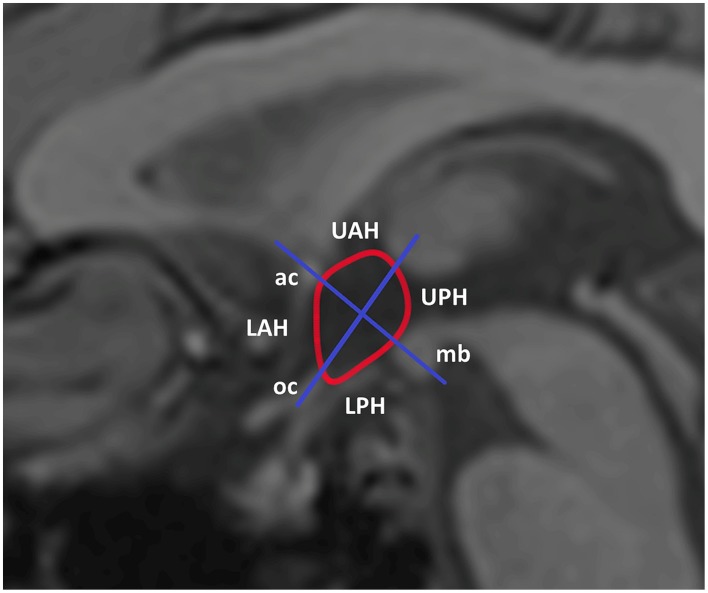
**Segmentation and subdivision of the hypothalamus into four ROIs**. For analysis of the hypothalamic BOLD response the hypothalamus was manually segmented using several anatomical landmarks. For further analysis the hypothalamus was divided in four spate subdivisions. LPH/UPH, lower/upper posterior hypothalamus; LAH/UAH, lower/upper anterior hypothalamus; mb, mammillary body; oc, optic chiasm; ac, anterior commissure.

Delineation of the hypothalamus region of interest (ROI) was performed manually, based on predefined anatomical borders with use of the anatomical image as an aid to delineate the following anatomic landmarks: anterior commissure, optic chiasm, and mammillary body. Two orthogonal axes were used to divide the hypothalamus into four sub regions as shown in Figure 1 according to Matsuda et al. (1999). In addition, an ROI was delineated in gray matter, superior of the genu of the corpus callosum, as a reference region. The mean signal within every ROI was established for each time point. The mean baseline signal for each ROI was calculated by averaging the signal over all measurements up to half a minute before drinking of the glucose solution started. To obtain the percentage signal change relative to baseline, the post-drink measurements were normalized to the mean baseline signal. The signal in the reference ROI was used to correct for scanner drift. To test for differences in average hypothalamic BOLD response before and after drinking the glucose solution participant data were pooled per time point. First a student's *t*-test was used to compare the baseline response between groups. A paired *t*-test was then used to compare the average BOLD response post-glucose ingestion to the average baseline response before glucose ingestion. To compare the BOLD response over time between patients and controls mixed model analysis was performed. General linear mixed model analysis was performed using study group as a fixed effect, time point as a covariate and participant as a random factor.

**Figure 2:**
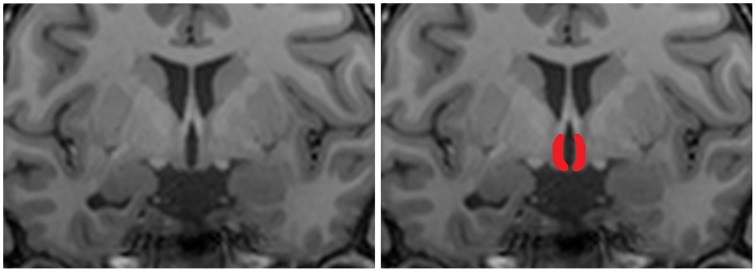
**Coronal T1 weighted image of the hypothalamus**. For structural analysis of the hypothalamus a region of interest was manually drawn on T1 scans. The left panel shows an representative coronal view of a T1 scans, the right panel shows an example hand delineated region of interest overlaid over the original image.

To analyze differences in hypothalamic and overall brain structure we performed manual segmentation of the hypothalamus to calculate hypothalamic volume and we performed VBM analysis to determine voxel-wise differences in volume between groups according to Holle et al. (2011). Manual segmentation of the hypothalamus was done based on morphological anatomical landmarks as described earlier (Goldstein et al., 2007; Klomp et al., 2012; Terlevic et al., 2013). A coronal view of an example segmentation of the hypothalamus is shown in Figure 2. Differences in hypothalamic volume between patients and controls were tested using an independent samples *t*-test.

For the voxel-wise comparison, standard VBM analysis was performed using the FSL-VBM tool (Good et al., 2001). To analyze the potential differences, voxel-wise general linear model (GLM) analysis was carried out, using permutation-based non-parametric testing, correcting for multiple comparisons across space (Nichols and Holmes, 2002).

## Results

### Population characteristics

Characteristics of patients and controls are shown in Table 1. Structural MRI data was obtained for all participants. As two patients resisted drinking the glucose solution at the last moment during the scanning session, valid fMRI data was obtained for 8 patients.

**Table 1:** **Participant characteristics**.

	**Patients (*n* = 10)**	**Controls (*n* = 11)**	***p*-value**
Age (years)	22.1 (3.3)	20.8 (0.52)	0.217
Weight (kg)	45.1 (4.06)	59.3 (3.05)	<0.001
BMI (kg/m^2^)	15.6 (1.02)	20.3 (1.5)	<0.001
Duration of AN (years)	3.54 (2.30)	NA	
AN subtype R/BP (%)	56/44	NA	

*Values are reported as means with SD (Standard Deviation); NA, not applicable; BMI, body mass index; kg/m^2^, kilogram per square meter; R, restricting; BP, binge-eating purging*.

### Functional hypothalamic response to glucose ingestion

**Figure 3:**
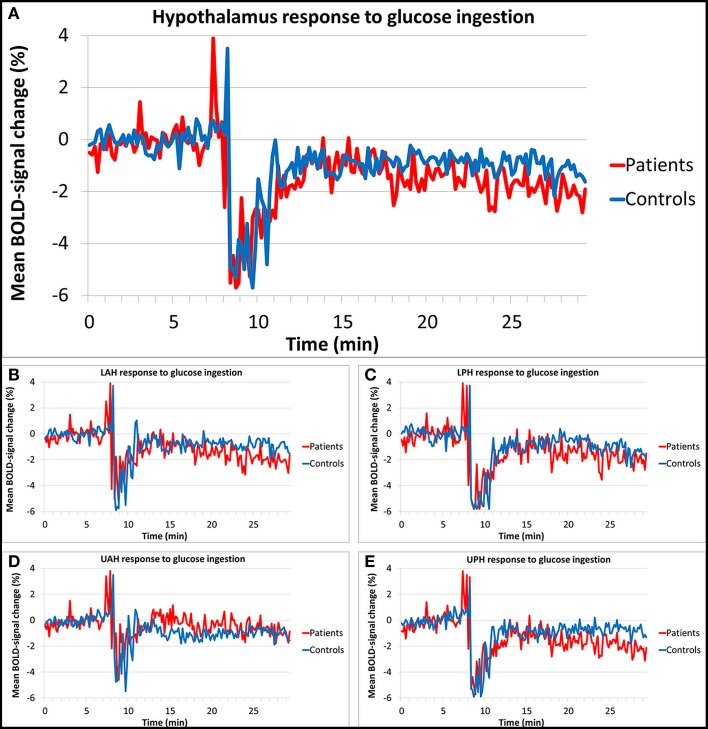
**Relative BOLD fMRI signal change of the hypothalamus in response to glucose**. The hypothalamic BOLD response showed a decrease of 1.4% in patients and a decrease of 1.0% in controls after glucose ingestion, no significant differences in response were found between groups. Segmentation of the hypothalamus into four subdivisions showed similar results. **(A)** Total hypothalamus, **(B)** lower anterior hypothalamus (LAH), **(C)** lower posterior hypothalamus (LPH), **(D)** upper anterior hypothalamus (UAH), **(E)** upper posterior hypothalamus (UPH).

Figures 3A–E show the results of the hypothalamic response to glucose ingestion for patients and controls, in the total hypothalamus and the four sub regions. The percentage BOLD-signal change from baseline value is shown over time, averaged per study group. At around 8/9 min a peak and drop in signal was observed, which is associated with head movement during drinking. The baseline response used for normalization was not significantly different between groups (average baseline signal in patient was 0.9327, SD 0.064 and 0.9017, SD 0.063 in controls, *p* = 0.89). Controls showed a significant average hypothalamic BOLD-signal change of -1.0% compared to the baseline in response to the oral glucose load. Patients showed a significant average signal change of −1.4% compared to baseline. There was no significant difference in total hypothalamic BOLD response between patients and controls (estimated difference between groups 0.4%, standard error 1.2%, *p* = 0.721). The LPH, LAH, and UPH segments showed similar responses that were not significantly different between groups either (estimated difference 0.4%, standard error 1.3%, *p* = 0.762, estimated difference 0.5%, standard error 1.4%, *p* = 0.736, and estimated difference 0.6%, standard error 1.3%, *p* = 0.634, respectively). In the UAH a somewhat different response to glucose ingestion was observed; here the response of the patients was smaller than in the other sub regions, however this was roughly the same for the controls (estimated difference 0.5%, standard error 1.0%, *p* = 0.601). In addition, no significant response difference in BOLD signal after glucose intake for each individual hypothalamic sub region was observed between subjects with AN and control subjects.

### Structural brain differences

**Figure 4:**
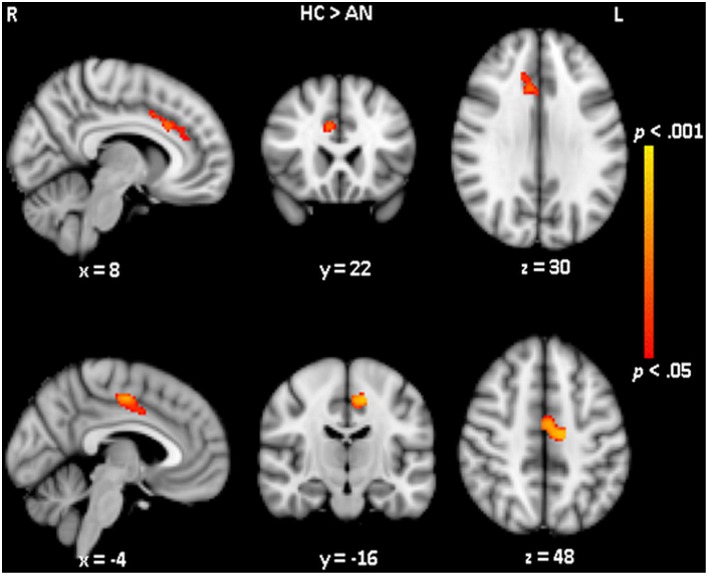
**Voxel-wise structural differences between healthy controls and anorexia nervosa patients**. Voxel based morphometry analysis showed a decreased gray matter volume of the cingulate cortex in AN compared to controls. The upper row shows the area of decreased gray matter in the right hemisphere the lower row shows the area of decreased gray matter in the left hemisphere.

No differences in hypothalamic volume was observed between the AN patients (mean hypothalamic volume of 0.482 cm^3^, SD 0.064 cm^3^) and healthy control participants (mean volume of 0.477 cm^3^, SD 0.057 cm^3^). VBM was used to assess voxel-wise differences in brain volume. VBM results revealed two clusters (one in each hemisphere) where the volume of the cortex was significantly different between the patients and controls (see Figure 4). Compared to controls, AN patients showed significantly (*p* < 0.05) reduced gray matter volume in the right cingulate gyrus, anterior division (number of voxels: 231; MNI x, y, z: 8, 22, 30) and reduced gray matter volume in the left cingulate gyrus, posterior division (number of voxels: 237; MNI x, y, z: −14, −16, 48).

## Discussion

The results presented in this study shows that the average hypothalamic BOLD response to glucose ingestion is not different between patients with AN and healthy controls. In addition we show that the hypothalamic volume is not different but the volume of the cingulate cortex, a higher-order cortical structure involved in cognitive processes, is decreased in AN.

To the best of our knowledge, this is the first study to investigate the direct response of the hypothalamus following glucose ingestion in patients with anorexia nervosa. The hypothalamus plays a key role in the intricate and complex neuroendocrine interactions that govern food intake and energy homeostasis. Glucose sensing neurons that are present in several hypothalamic nuclei communicate with other neuronal systems involved in these intricate processes. Receptors that are responsive to several neurotransmitters involved in food intake are present in the hypothalamus as well (King, 2006). In addition, the hormones leptin and insulin signal the hypothalamus to reduce food intake (Niswender and Schwartz, 2003). The levels of these neuropeptides become abnormal with weight loss, signaling the body that it needs food, as there is not enough fuel to maintain body processes. Despite the fact that these levels are abnormal in AN patients, (Smitka et al., 2013) they still manage to refrain from eating, indicating that AN patients might be overriding the hypothalamic signaling.

In healthy participants glucose ingestion decreased hypothalamic neuronal activity (Smeets et al., 2005a, 2007; Vidarsdottir et al., 2007). We observed a similar decrease in overall hypothalamic neuronal activity after glucose ingestion in the AN patients compared to the healthy controls. The AN patients showed a slightly lower response on average, but this difference was not statistically significant. Within the four hypothalamic subdivisions no significant differences were detected either, indicating that more specific areas in the hypothalamus also showed no significantly different response between groups. The observed glucose responses are in line with previous hypothalamus glucose studies in healthy controls. Vidarsdottir et al. (2007) observed signal decreases up to 3%, and Smeets et al. (2005b) observed decreases up to 2.5%. However, since these studies both used 75 grams of glucose instead of the 50 grams used in the present study, the observed signal changes are slightly higher, as the response of the hypothalamus to glucose is dose-dependent (Smeets et al., 2005a). In addition, the observed decreases in BOLD signal are within the expected measurable range with 3.0 Tesla MRI. At a field strength of 3.0 Tesla, a 3% change in BOLD signal is estimated to be the maximal BOLD effect that can be measured (van der Zwaag et al., 2009). This indicates that we measured a true effect size, as the mean signal changes were within this 3% range. An earlier study by Holsen et al. assessed the hypothalamic activity in AN patients in response to pictures of high-calorie food objects in a fasted and satiated state (Holsen et al., 2012). When comparing hypothalamic activity changes between AN patients and the control group this study did not report any significant differences, both in a fasted and a satiated state. On the other hand, when fasted activity changes were analyzed within the different groups, a significant hypothalamic activation was found in the controls, but not in the AN patients. When comparing this study to ours, Holsen et al. assessed cognitive processes, as food pictures trigger neural responses that activate appetite regulating regions including the hypothalamus, (van der Laan et al., 2011) whereas we assessed the physiological hypothalamic response, as an oral glucose load actually changes blood glucose levels, signaling the glucose sensing neurons in the hypothalamus (Levin et al., 2004). Together these findings indicate that in AN direct hypothalamic nutrient sensing is not different but higher-order cognitive processes might indeed be influencing and/or suppressing the hypothalamic signaling.

Our structural findings are in line with the intact function of the hypothalamus in response to energy intake as we did not find an different hypothalamic volume in the AN patients by manual segmentation and voxel based analysis compared to healthy control subjects. However, the VBM analysis did reveal significantly smaller volumes in parts of the cingulate cortex in the AN patients (bilaterally). The cingulate cortex is a region known to be involved in emotion, attention, pain, and memory (Torta and Cauda, 2011). This combination of processes gives the cingulate cortex an important role in various psychiatric disorders, such as schizophrenia and major depressive disorder (Davey et al., 2012; Yan et al., 2012). In a recent systematic review about the neurobiology of AN, it was shown that differences in cingulate cortex structure and function are often observed in AN as well (Phillipou et al., 2014). Using VBM, an earlier study observed decreased gray matter volume in the anterior cingulate cortex in acute AN patients compared to healthy controls, and also in long-term weight-restored AN patients compared to healthy controls (Friederich et al., 2012). A further study used resting state brain activity to assess intrinsic connectivity within the cerebellar network in AN and bulimia nervosa patients. In AN patients, the cerebellar network was more connected with the posterior cingulate cortex compared to healthy controls, whereas BN patients showed greater connectivity in the anterior cingulate cortex compared to both healthy controls and AN patients (Amianto et al., 2013). These and many more studies indicate that the cingulate cortex may be involved in the pathophysiology of AN.

A technical limitation of our study is that the hypothalamus is a complex region for automated segmentation due to the inherent lack of contrast on T1-w and T2-w MRI. Therefore, we segmented the hypothalamus manually by using clear anatomic landmarks: anterior commissure, optic chiasm, and mammillary body. These landmarks were used for functional and structural analysis. Possible small variations in size between AN patients and controls are difficult to detect using this manual segmentation, especially on the spatial resolution available with MR imaging. Additionally, the available spatial resolution of the BOLD fMRI also prevents us to further determine which specific nuclei in the hypothalamus respond to the glucose ingestion. Therefore, we cannot conclude that there are no differences in activity between AN patients and controls in all nuclei of the hypothalamus but we can only conclude that the overall neuronal activity in the hypothalamus is unaffected. A further potential limitation of the present study is that it was not designed as a randomized oral water/glucose crossover design. To limit the study load especially for anorexia nervosa patients, we have chosen not to use a water condition as an additional control reference. It should be realized that the intrinsic reference values, or calibration, of our experiments is the BOLD signal before glucose administration (i.e., the BOLD signal in the first 8 min). In the AN patients the anticipation of the glucose ingestion could perhaps influence this baseline BOLD signal due to increased stress. However, our analysis of the baseline signal between groups shows that the signal level is comparable between patients and controls, indicating that an external water control is not necessary to compare the relative BOLD response between groups. In addition, several experiments in both control subjects and patients with type 2 diabetes have clearly shown that water in itself without glucose does not elicit a BOLD response and would add very little information (Matsuda et al., 1999; Smeets et al., 2005a, 2007; Vidarsdottir et al., 2007).

A general major limitation and methodological issue in all AN research is the question of cause and effect between the impact of malnutrition on neurobiological factors and pathological eating. Therefore, caution is warranted when interpreting research findings in AN patients. A possible strategy to avoid the confounding effects of abnormal nutrition is studying weight-restored AN patients (Kaye et al., 2013). However, structural differences of the cingulate cortex have also been observed in weight-restored AN patients, (Muhlau et al., 2007; Friederich et al., 2012) indicating that these findings are not merely a consequence of malnutrition. The fact that we did not observe other structural differences that are often reported, such as the amygdala, hippocampus and overall reduced gray matter volumes, might be explained by our rather heterogeneous patient group regarding AN subtype and duration of disease at the time of participation (Brooks et al., 2012). Despite this heterogeneity we did observe decreased cingulate cortex volume, indicating that this region plays a key role in the pathophysiology of AN. Our study used a modest sample size of participants, mostly caused by the difficulty of recruiting AN patients willing to participate in the drinking of the glucose solution. Our sample size could be a problem in detecting subtle differences in hypothalamic response between patients and controls. However, several other case-control studies using the same fMRI technique for determining hypothalamic function have detected significant differences using similar sample sizes (Matsuda et al., 1999; Vidarsdottir et al., 2007). In addition, power calculations for our study showed that each group would need to consist of 23 participants. Despite of our small sample size we do find significant structural differences, further suggesting that the effect size, and possibly the pathophysiological role, of structural differences is much larger than functional differences.

### Conflict of interest statement

The authors declare that the research was conducted in the absence of any commercial or financial relationships that could be construed as a potential conflict of interest.
